# Treatment of Dental Pulps

**Published:** 1868-06

**Authors:** 


*Treatment of Dental Pulps.*—Dr. W. H. Atkinson at a meeting of the Brooklyn Dental Association gave the following method of treatment :

"When a pulp is exposed and aches, I remove all extraneous matter from the part ; if this does not arrest the pain I dry out the cavity by applying pieces of bibulous paper until it is perfectly dry.

I then apply pure creosote and hold a dry napkin around the tooth for a minute, and if the pain continues I remove the napkin and syringe the cavity with tepid water, washing out all the creosote, and then apply a fresh napkin and proceed as before; then apply the best chloroform upon a pledget of cotton.

If this does not succeed repeat it again, and apply the tincture of aconite. In most cases it will not be necessary to repeat this process. I next proceed to prepare the cavity for the filling. I apply the napkin, or sheet rubber to keep dry and then delicately, fragments of bibulous paper until all moisture is removed, when I place a drop of creosote on the exposed part and then put a soft mass of osteo-plastic over the creosote.

I wait for the osteo-plastic to firmly set, then remove the excess and fill boldly."

